# Giant Ileocecal Inflammatory Fibroid Polyp: Unique Clinical Presentation

**DOI:** 10.1155/2020/8811404

**Published:** 2020-07-21

**Authors:** Hatim Almaghrabi, Murouj Almaghrabi, Haneen Al-Maghrabi

**Affiliations:** ^1^College of Medicine, Umm Al-Qura University, Makkah, Saudi Arabia; ^2^Department of Pathology and Laboratory Medicine, King Faisal Specialist Hospital and Research Center, Jeddah, Saudi Arabia

## Abstract

Inflammatory fibroid polyps (IFPs) are infrequent gastrointestinal tract benign neoplasms. They mostly occur in the stomach especially the antrum. Signs and symptoms depend on their anatomic location and lesion size. Lesion biopsies are very challenging for accurate diagnosis in these lesions. Histopathological examination of resected tissue along with immunohistochemical studies is the perfect way to confirm the final diagnosis. In this paper, the authors present an unusual case of IFP in a 23-year-old female patient presented to the emergency room (ER) with a picture of intestinal obstruction and severe abdominal pain. Her investigations revealed a huge mass located at the ileocecal valve, with overall features mimic gastrointestinal stromal tumor (GIST) clinical presentation. Surgical resection is considered the most commonly used treatment method. The overall prognosis is good with a low risk of recurrence.

## 1. Introduction

Inflammatory fibroid polyp (IFP) was first described in 1920 by Konjetzny as polypoid fibroma [1]. Later in 1949, Vanek described 6 cases of gastric lesions named as gastric submucosal granuloma with eosinophilic infiltration [2]. Helwig introduced the term inflammatory fibroid polyp in 1953 [3]. The exact etiological cases and underling pathogenesis are not well understood yet. IFP is an uncommon benign neoplasm commonly located at the antrum part of the stomach [4]. IFP can be associated clinically with hypochlorhydria or achlorhydria [5]. Radiological and endoscopic studies usually revealed a sessile or pedunculated polypoid mass. Due to its rarity and nonspecific signs and symptoms, correct perioperative diagnosis is usually difficult. Histopathologic examination revealed characteristic bland cellular submucosal lesion composed of fibroblastic and vascular channels proliferation. This bland cellular proliferation is arranged in a whorl-like around blood vessels [6]. This appearance should be distinguished from other spindle cell lesions, most importantly GIST. Surgical resection remains the mainstay method of treatment for giant large colonic IFPs. Endoscopic resections are very useful in smaller pedunculated polyps. Due to the benignity of IFPs, they tend to have low postresection recurrence rate. Acero and colleagues studied 26 cases of IFPs from 25 patients, in which 16 cases are located in the antrum, 7 located in the ileum, 2 in the jejunum, and one in the colon. All of them show no evidence of recurrences after mean length of follow-up was 60.6 months [7]. Kayyali and colleagues reported 32 cases of colonic IFPs. In which 15 cases of them are in the cecum (44%), 3 cases in descending colon, 3 in ascending colon, 8 in the transverse colon, 2 in sigmoid, and one in the rectum. These cases were treated by surgical resection in 20 cases (58%) and endoscopic resection in only 8 (23%). All of them show no evidence of colon recurrence [8]. However, the literature reported 2 cases of IFP recurrence located in the small intestines [9, 10], and one case located in the stomach [11].

In this paper, we present a unique case of large IFP involving the ileocecal junction in a young female patient who presented to the ER with intestinal obstruction pictures. Her detailed clinical presentation, hospital course, investigations, and management with more detailed differential diagnosis are discussed below.

## 2. Case Presentation

**Figure 1:**
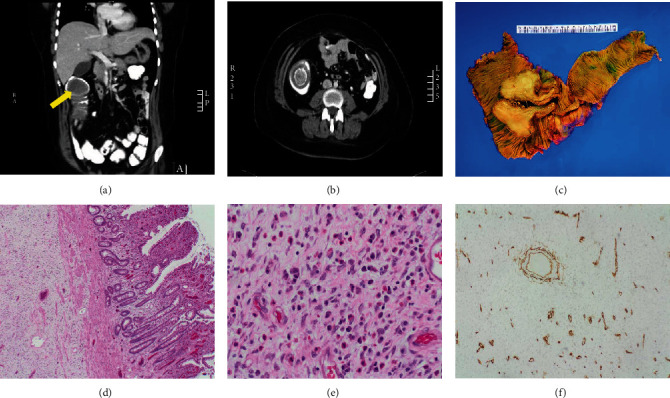
Representative hematoxylin and eosin (H&E) and immunohistochemical staining of inflammatory fibroid polyp (IFP). (a) Coronal (yellow arrow on mass). (b) Axial CT scan with contrast revealed ileocolonic intussusception measuring in maximum longitudinal axis about 7.4 cm. (c) Gross examination revealed a large pedunculated polypoid mass measuring 7.5 cm with attached stalk measuring 5 cm, located at the ileocecal valve. (d) Submucosal mass characterized by spindle cells embedded in a loose myxoid matrix (H&E; 2x). (e) Heavy inflammatory infiltrate with eosinophils and mast cells are observed (H&E; 20x). (f) CD34 expressed prominently in blood vessels, while tumor cells were negative (4x).

A 23-year-old female patient with known case of multiple comorbidities, systemic lupus erythematosus (SLE), antiphospholipid syndrome, venous thromboembolism, old DVT, pulmonary hypertension, systemic hypertension, and vitamin B12 and D deficiencies is presented. She was controlled on her medications. She presented to the ER with a picture of intestinal obstruction complaining of a history of severe abdominal pain, sharp, progressive, and continuous, associated with nausea, vomiting, and diarrhea for the past 2 days since her presentation. At the time of admission, the patient looked ill, oriented, in pain about 3-4 out of 5 pain scale. Abdominal examination revealed a slight distended, mild tender abdomen in the right iliac fossa. There were organomegaly or palpable masses. Laboratory data showed no anemia, no elevated blood eosinophil ratio, nor abnormal globulin fraction. A computed tomography (CT) scan with contrast revealed ileocolonic intussusception measuring in maximum longitudinal axis about 7.4 cm with large polypoid soft tissue mass acting as a leading point measuring about 7 cm in its maximum dimension (Figures 1(a) and 1(b)). Differential diagnosis included GIST and adenomatous polyp. The patient was transferred to the surgical department for urgent surgical consultation. The patient underwent right hemicolectomy which was done for the patient smoothly without postoperative complications. The resected specimen was sent for a histopathology examination. Under prober opening and overnight formalin fixation, the specimen showed large pedunculated polypoid mass measuring 7.5 × 4 × 2 cm with attached stalk measuring 5 cm, located at the ileocecal valve (Figure 1(c)). Cut sectioning of the polyp revealed a homogenous white fibrous area. The rest of the colon and ileum are grossly unremarkable. Hematoxylin and eosin- (H&E-) stained slides revealed large well-defined submucosal mass composed of bland spindle cell lesion proliferation concentrically arranged around thick-wall blood vessels (forming onion skinning appearance) (Figure 1(d)). Background of edematous changes with mixed inflammatory cells infiltrates particularly the eosinophil were seen (Figure 1(e)). No evidence of atypical cells, high nuclear to cytoplasmic ratio, or mitosis was seen. Immunohistochemistry studies showed positive vimentin stain. CD34 (Figure 1(f)) and CD117, discovered on GIST (DOG-1), Desmin, Caldesmon, muscle-specific antibody (MSA), smooth muscle antibody (SMA), beta-catenin, anaplastic lymphoma kinase (ALK-1), signal transducer and activator of transcription 6 (STAT6), neurofilament, cytokeratin-pan, and S-100 were all negative. Overall histopathology examination and immunohistochemistry confirmed the diagnosis of IFP. At the time of discharge, the patient was stable with a colostomy bag. A few weeks later, the patient followed up in the clinic with healthy and stable conditions. Five years later, the patient was healthy and shows no evidence of recurrence.

## 3. Discussion

IFPs are uncommon benign mesenchymal lesions. IFPs are distributed almost equally between male and female populations. Some suggested that it shows a slight female predominance. The mean age of presentation is 41 years old (average between 19 and 46 years old) [12]. IFP range in size from few millimeters to multiple centimeters. IFPs are frequently occuring in the gastric antrum (66-75%), ileum (20%), duodenum (1%), colon (7%), esophagus (1%), gall bladder (1%), and rarely appendix (less than 1%) [13, 14]. However, Liu and colleagues [15] studied a large series of IFPs (83 cases) which revealed that the most common site of involvement is the large bowel (37%), then followed by the gastric antrum (23%) and small bowel (20%). Most IFPs are asymptomatic and found incidentally during endoscopic evaluation for other medical conditions. One study showed that 83.3% of IFP patients presented with clinical heterogeneous signs and symptoms, usually nonspecific, depending on tumor size and anatomic location [12]. Most reported cases are presented with abdominal pain (54%) and anemic manifestation (17%) commonly due to bleeding (33%) [16]. As they increase in size, they can lead to hematochezia, weight loss, diarrhea, or intussusceptions. One case document an IFP patient presented with hypovolemic shock [17]. The underlying etiology is not well understood; some believe that it is due to an exaggerated human body response to allergy after prolonged inflammatory stimulant. Others suggested that it is a response to irritation such as trauma, chemical injury, tuberculosis, sarcoidosis, and helicobacter pylori infection. IFP was reported in a few cases of patients who had autoimmune diseases such as Crohn's disease, rheumatoid arthritis, SLE, and ankylosing spondylitis. These findings raise the possibility of immunological contributing factors to IFP pathogenesis. Yet, no supportive clinical evidence found in the literature between collagen disease and IFP development [16, 18]. IFP used to be a reactive nonneoplastic tumor-like condition until the detection of activating platelet-derived growth factor receptor alpha (PDGFRA) mutation in 2008 in 70% of cases [4]. This confirms the clinical evidence of IFP neoplastic pathogenesis by activating mutation. However, no clinical evidence or medical data of tumor invasiveness, aggressive clinical behavior, or metastasis is reported to the best of our knowledge. PDGFRA gene mutation is commonly described in various body tumors. Most importantly, GISTs are associated with activating receptor tyrosine kinase signaling mRNA pathway. This mutation has a strong genotype and clinical correlation with tumor morphology, behavior, and response to therapy with imatinib. Moreover, PDGFRA is found in idiopathic hypereosinophilic syndrome caused by 4q12 deletion chromosome which results in FIP1L1-PDGFRA gene fusion. Clinical studies provided that activating PDGFRA mutation found in IFPs have no biological or genomic association with PDGFRA-mutated GIST pathways. GIST can have different clinical behavior and some cases can show recurrence and metastasis, whereas IFPs do not metastasize. Thus, the genomic behavior of IFPs and GISTs is different. Also, patients with IFP do not usually suffer from signs and symptoms of systemic hypereosinophilia, which explains the absence of FIP1L1-PDGFRA translocation in IFPs. PDGFRA gene mutation in IFP provides an example of a tumor with somatically acquired mutation alterations [19]. Thus, IFPs must be differentiated from other gastrointestinal tract spindle cell lesions. A definitive diagnosis is made by histopathology examination and immunohistochemistry studies. IFPs can present with different histologic background, classical, cellular, myxoid, or sclerotic. Most of IFPs are presented as submucosal lesions contain spindle cell and stellate stromal cells. Perivascular spindle cell proliferation forming “onion skinning” appearance is present in about half IFPs. A mixture of inflammatory cells infiltrate is seen typically dominated by eosinophils. Minimal or no mitotic activities are seen. IFPs are commonly reacted positively for vimentin and CD43 (prominently in blood vessels network), while negative for Desmin, SMA, MSA, S-100, and CD117 [17, 20]. Therapeutic management of IFPs can be managed similarly to other benign polyps. Theses polyps cannot be evaluated probably with gross examination along. It should be sent for histopathology examination to confirm tumor benignity and to rule out the presence of underlining low- or high-grade dysplasia. The management plan should be based on the patient presentation and expertise of a gastroenterologist physician. Patients with chronic pain or signs and symptoms of obstruction should be managed by surgical resection. Patients with a clinical presentation of acute bleeding should be adequately hemostasis managed and lesion localized; once they are stabilized, they can undergo polyp resection. Histopathology examination and immunohistochemistry analysis must be done to confirm the final diagnosis and to exclude any dysplasia/malignancy evidence. Due to IFP benign potentials, routine endoscopic follow-up is not usually indicated for asymptomatic patients [21]. The differential diagnosis includes GIST, schwannoma, leiomyoma, inflammatory myofibroblastic tumor (IMT), and plexiform fibromyxoma. GIST is the most common mesenchymal gastrointestinal tract tumor, which can be spindle, epithelioid, or mixture of the two patterns. GIST would react positively to CD117, CD34, or DOG1 [22]. Schwannoma is usually a neural tumor origin that is diffusely stained for S-100, arising from muscularis propria, with characteristic schwannoma hyalinized thick blood vessels with peripheral lymphoid cuffing [23]. Leiomyoma is a benign smooth muscle neoplasm; grossly, it reflects a whorled white-tan bulging cut surface that is composed of a fascicular (whorled) pattern of smooth muscle bundles growth which reacted to SMA, MSA, Desmin, and h-caldesmon positively. IMT is a spindled myoepithelial cell proliferation with lymphocytic infiltrate that mostly stained with ALK-1. Plexiform fibromyxoma is usually a multinodular concentric around muscularis propria which lacks blood vessels and negative for CD34 [24].

## 4. Conclusion

To sum up, we present a case of unique clinical presentation as a giant ileocecal mass causing severe abdominal pain and clinical picture of ileocolonic intussusception, with clinical differential diagnosis of GIST. IFP is an unusual tumor of the ileocecal area and represents a common polyp in the gastric antrum and rectosigmoid colon. The underlying pathogenesis is still not well developed; however, PDGFRA activation is proved in these benign neoplasms. Patients are usually presented with vague sign and symptoms which are not specific. Thus, histopathological with the aid of immunohistochemistry studies is a must to confirm the diagnosis.
